# Micronized Recycle Rubber Particles Modified Multifunctional Polymer Composites: Application to Ultrasonic Materials Engineering

**DOI:** 10.3390/polym14173614

**Published:** 2022-09-01

**Authors:** Vicente Genovés, María Dolores Fariñas, Roberto Pérez-Aparicio, Leticia Saiz-Rodríguez, Juan López Valentín, Tomás Gómez Álvarez-Arenas

**Affiliations:** 1 Instituto de Tecnologías Físicas y de la Información, Spanish National Research Council (CSIC), 28006 Madrid, Spain; 2 Signus Ecovalor, S.L., 28033 Madrid, Spain; 3 Instituto de Ciencia y Tecnología de Polímeros, Spanish National Research Council (CSIC), 28006 Madrid, Spain

**Keywords:** multifunctional composites, particle loaded polymers, ultrasonic transducers, ultrasonic materials, damping in composite materials, complex elastic moduli of composites, ultrasonic testing, recycled end-of-life tires rubber

## Abstract

There is a growing interest in multifunctional composites and in the identification of novel applications for recycled materials. In this work, the design and fabrication of multiple particle-loaded polymer composites, including micronized rubber from end-of-life tires, is studied. The integration of these composites as part of ultrasonic transducers can further expand the functionality of the piezoelectric material in the transducer in terms of sensitivity, bandwidth, ringing and axial resolution and help to facilitate the fabrication and use of phantoms for echography. The adopted approach is a multiphase and multiscale one, based on a polymeric matrix with a load of recycled rubber and tungsten powders. A fabrication procedure, compatible with transducer manufacturing, is proposed and successfully used. We also proposed a modelling approach to calculate the complex elastic modulus, the ultrasonic damping and to evaluate the relative influence of particle scattering. It is concluded that it is possible to obtain materials with acoustic impedance in the range 2.35–15.6 MRayl, ultrasound velocity in the range 790–2570 m/s, attenuation at 3 MHz, from 0.96 up to 27 dB/mm with a variation of the attenuation with the frequency following a power law with exponent in the range 1.2–3.2. These ranges of values permit us to obtain most of the material properties demanded in ultrasonic engineering.

## 1. Introduction

Two-phase composites made of a polymeric matrix reinforced with particulate matter are widely used in quite diverse fields and applications as the addition of different filler materials and filler volume fractions has proved to be an efficient way to tailor optical, acoustical, mechanical, dielectric, magnetic and thermodynamic properties [1]. A wide variety of particulate fillers has been used [1]: metals (tungsten, iron, etc.), semimetals (silicon), oxides (alumina, cerium oxide, silicone oxide, titanium oxide, zirconia, etc.), ceramics (barium strontium titanate, PZT, silicon carbide, etc.), polymers (elastomers, rubbers [2,3], resins, etc.) and other more complex materials like different recycled materials (carbon black, fly ash, toner waste, bio-agricultural waste, and recycled rubber granulates and powders from end-of-life tires [4,5,6,7]). There is also growing interest and applications for the case of nanoparticle-loaded polymers (see [8] for a review).

Applications of particle-loaded polymers are as numerous as the different types of materials mentioned above [9]. Examples can be found in the design of high damping materials [10], in the construction industry and in the fabrication of precision machine tools [5], in the fabrication of electronic components for encapsulation and EMI shielding [11] and in aerospace applications or for automobiles, ships, and different electronic devices (where these composites are used to improve brittleness and impact strength of epoxy resins [3]). Rubber-reinforced composites have been used for improving mechanical properties in applications such as sound absorbing panels and impact protection slabs [3]. Hollow glass spheres are used to increase thermal and acoustic insulation of paintings and coatings, and to produce buoyancy materials [12]. Recycled rubber granulates and powders from end-of-life tires (ELT) also have well-established applications in the industry such as sport surfaces, road safety elements, modification of bituminous mixtures, insulation, brake pads or even for the manufacture of new tires. However, new applications for ELT-derived products are still demanded [13,14]. Recent developments in recycling processes open up new perspectives for ELT rubber. In this way, cryogenic milling allows reducing the particle size to about 100 μm, then, the resultant micronized rubber can be used in high added value applications.

In the ultrasonic field, these composites are normally called 0-3 connectivity composites [15], or 0-3/3-3 in the case of composites with very high particle volume fraction [16,17]. They are used in two different applications: (i) as components of piezoelectric transducers, either as active (piezoelectric) materials [18], or as passive materials (mainly backing blocks and matching layers [19]) to further extend the functionalities of the piezoelectric component (in terms of bandwidth, resolution and sensitivity), and (ii) to produce phantoms of human tissues for testing of ultrasonic image systems and procedures for medical applications. The design and manufacture of active 0-3 connectivity piezocomposite materials for ultrasonic transducers have been widely investigated [20,21,22,23]. This allowed achieving lower impedance piezoelectric composites, and ultrasonic transducers with larger bandwidth, or higher center frequency [24], as well as piezoelectric paints [25] and cements [26]. Passive 0-3 connectivity composites have been largely used to produce damping backing blocks [27,28,29,30,31,32,33,34] or matching layers, where the use of nanoparticles allow for the possibility to make matching layers for high frequency applications [35,36]. Sometimes, the use of two different types of particulate matter has also been reported [29,37]. Another use of passive 0-3 connectivity composites is to produce human tissue phantoms (with reduced cost, longer lifetime, and able to replicate more complex anatomical structures) that contribute to facilitated advances in ultrasonic diagnostic and therapeutic strategies [38,39].

In general, in these types of applications, it is necessary to precisely control the acoustic impedance of the composite, the ultrasonic damping and the contribution of the scattering. In the case of backing blocks, a large impedance is normally required (so that the vibration in the piezoelectric element can be damped and hence the bandwidth of the transducer enlarged, and the axial resolution improved), while a large attenuation and a very reduced backscattering is required to reduce noise in the transducer and eliminate any echo from the backing block back surface. In the case of impedance matching layers, impedance must be precisely tuned to an intermediate value between that of the piezoelectric component and the insonicated medium, while attenuation has to be minimized. Finally, for tissue mimicking phantoms, it is necessary to tune the impedance, the attenuation coefficient and its variation with the frequency and the backscattering of the material to that of the tissues. A similar problem is found in the design of damping composite materials, where two functionalities are involved: the load capability and the damping efficiency, and where the proposed figure of merit is the product |E*|tan δ, where |E*| is the modulus of the complex elastic modulus (E*) and tan δ = Im(E*)/Real(E*) [10]. 

Several strategies have been attempted to increase the damping, to control the scattering in composite materials at ultrasonic frequencies and to decoupled damping and impedance modifications. They can be classified in three main groups depending on the composite component that is modified to obtain the desired properties: (i) the particles, (ii) the matrix, and iii) both. Lutsch (1962) [27] introduced large rubber particles achieving a moderate attenuation (up to 0.8 dB/mm at 1 MHz), with intermediate acoustic impedance (~8 MRayl). Similarly, Ju-Zhen [29], added cerium oxide particles to tungsten-loaded epoxy to increase attenuation. Scattering by the filler particles in the backing block can be enhanced by increasing the particle concentration or the mean particle size or the impedance mismatch between the particles and the matrix, but this must be done carefully as the backscattering can be an unacceptable source of noise. Cho et al. [40] proposed a fabrication process that permits the increase of the load of particles, achieving attenuation values between 3 to 5 dB/mm at 3 MHz, with acoustic impedance between 4 and 4.6 MRayl.

Increase of the composite damping by using more attenuating polymer matrices can be achieved by either using a different polymer or by modifying the properties of the selected one (by adding a plasticizer, by blending different formulations, or by lowering the cross-link density [3]). In this sense, State et al. [37] achieved attenuation values between 35 and 40 dB/mm at 8 MHz, and an almost linear variation with frequency, for tungsten and alumina-loaded polyurethanes (compared with reference values of about 19 dB/mm at 8 MHz for similar alumina-loaded epoxy composites), with impedance of about 2.6–3.3 MRayl. 

El-Tantawy and Sung [41] tried a combined approach: large Ti particles, and a modification of the polymer by adding a plasticizer (glycerol) and a coupling agent (silane). Achieved attenuation values were between 2.2–3.9 dB/mm at 3 MHz, with acoustic impedances between 2 and 7.8 MRayl. Another approach consists of adding liquid rubber [42,43] that has also been used to reinforce the brittle character of thermosetting epoxies [3]. There are two different ways for the modification of the composite properties by adding liquid rubber. In the first case, rubber-epoxy separation occurs during the epoxy solidification giving rise to rubber domains, mainly as a result of the decrease in configurational entropy due to the increase in molecular weight as the epoxy cures [3]. In the second case, this separation does not take place [42]. In the case where rubber domains appear in the composite, impedance decreases (from 11.7 MRayl to 8.7 MRayl), however, attenuation dramatically increases, from 1 dB/mm to 6 dB/mm at 2 MHz. The advantages are that the viscosity increase of the epoxy + rubber mixture is moderate; this allows for a high load of particles and the obtained attenuation values are very high.

In this paper we analyze the possibility of tailoring, in a decoupled way, the load capability (determined by the composite impedance), the ultrasonic attenuation coefficient, its variation with the frequency, and the contribution of scattering of particle polymers by using two different types of particles: small particles of heavy metal (intended to composite impedance, with reduced scattering and increase EMI shielding) and micronized rubber powder from ELTs (intended to increase attenuation with reduced scattering and reduced impedance modification). A fabrication route compatible with transducer manufacturing is also proposed as this can be one of the applications of these composites: to enhance the functionality of the piezoelectric layer in piezoelectric transducers (in terms of bandwidth, resolution and sensitivity). This involves avoiding high temperatures and pressures in the fabrication of the composites that may compromise the piezoelectric response of the active component of the transducers. Finally, a modelling approach to calculate the complex elastic constants of the composites, the variation of the attenuation with the frequency and the relative contribution of the scattering is also proposed.

## 2. Materials and Methods

### 2.1. Materials

#### 2.1.1. Raw Materials

The employed raw materials are summarized in Table 1. 

Disk samples with a 30 mm diameter and thickness between 1.7 and 4 mm (Table 2) were manufactured following the procedure described in Section 2.2.2. In addition, a rubber plate sample was also produced by press molding of the ELT rubber powder at 160 °C and 200 bar for 30 min. Density is worked out from weight and size (disk diameter and thickness) measurements. 

The properties of these materials are shown in Table 3; in this case, the method to measure these properties is the one used to measure the composite samples. This method is described in Section 2.2.2. To account for the variation of the ultrasonic attenuation (α) with the frequency (*f*), we used a power law: $\alpha =\alpha_{0}{\left(f/f_{0}\right)}^{n}$. The exponent “*n*” is also listed in Table 3. 

**Figure 1:**
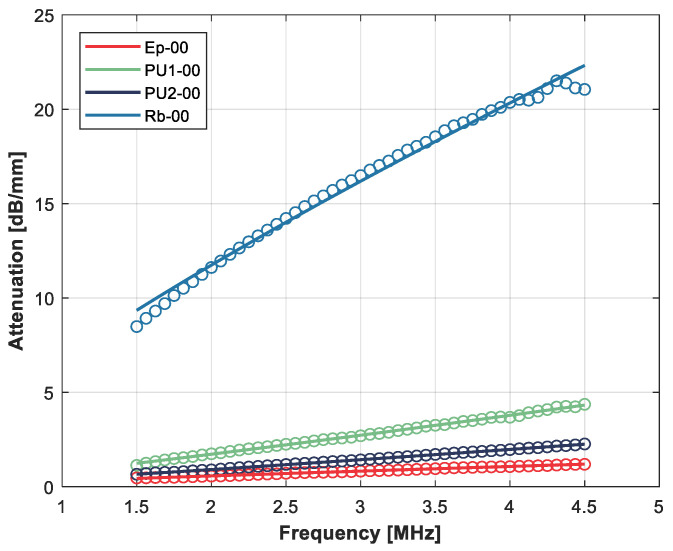
Variation in the ultrasonic attenuation coefficient with the frequency for the raw materials. Open circles: measurements. Solid line: fitting using a power law.

Given the novelty in the use of recycled rubber from ELT for ultrasonic applications and the lack and variability of ultrasonic data (in particular for “*n*”) for polymers, Figure 1 shows the measured attenuation versus frequency and the fitting with the proposed power law that permits the estimation of the exponent “*n*”.

Finally, Table 4 summarizes the main properties of the tungsten powder.

#### 2.1.2. Composite Material Samples

Following the methods described in Section 2.2.1, several samples were produced (see Table 5, Table 6, Table 7 and Table 8). The mass fraction of each component used in the mixture ($\mu_{i}$) is defined a (Equation (1)):

(1) $$\mu_{i}=m_{i}/\sum_{i=1}^{N}m_{i},$$

where *m_i_* is the mass of each component in the mixture. This mass fraction data together with the density of each component ($\rho_{i}$) is used to calculate the volume fraction of each component (Equation (2)):

(2) $$\phi_{i}=\left(\mu_{i}/\rho_{i}\right)/\sum_{i=1}^{N}\mu_{i}/\rho_{i},$$

Hence, we calculate the nominal density of the final composite ($\rho_{comp}^{*}$) (Equation (3)):

(3) $$\rho_{comp}^{*}=\sum_{i=1}^{N}\phi_{i}\rho_{i},$$

This nominal density is compared with the actual density ($\rho_{comp}^{}$) of the fabricated samples, which is obtained from the sample dimensions (diameter and thickness) and mass. From this comparison we work out the density deviation, (Equation (4)):

(4) $$Density\;deviation\;\left(\%\right)=\frac{\rho_{comp}^{}-\rho_{comp}^{*}}{\rho_{comp}^{*}}\times 100,$$

Table 5 describes the fabricated rubber-powder-loaded epoxy resin samples. The purpose of this series is to confirm the capability to efficiently fabricate these samples with the proposed method, to verify that the attenuation coefficient increases with the rubber load while scattering is not significantly increased, and to check the efficiency of the proposed model to predict the attenuation in these composites. The volume fraction ranges from 2% up to 35%. Just for comparison purposes, it can be mentioned that the rubber concentration values employed for epoxy toughening are, normally, 5–25 wt% [3]. The thickness of the sample has no relation with the composition and depends on the mold used (we used different molds with the same diameter but different height) and the degree of polishing. 

Table 6 summarizes the fabricated tungsten + rubber-loaded epoxy samples. Two series of samples have been produced, with low and high tungsten load, respectively. The first series is intended to obtain composites with acoustic impedance around 5 MRayl. In this case, the load of tungsten particles is reduced (about 15%), hence it is possible to add a relatively large amount of rubber load (up to 40%). The second series is intended to obtain composites with acoustic impedance close to 17 MRayl. In this case, the load of tungsten particles is larger (38–50%) so that it is only possible to add a more reduced amount of rubber load (up to 12%). The limit of the maximum particle load is determined by the epoxy viscosity, the pot life, the particle size and shape and the mixing technique.

For comparison purposes, some samples of tungsten-loaded epoxy resin and tungsten-loaded polyurethane were also fabricated and tested. These samples are described in Table 7 and Table 8.

#### 2.1.3. Equipment Used

Samples were weighed using a precision analytical laboratory balance, Nahita Blue, diameter of the samples was measured using a caliber and the thickness was measured using a micrometer (Mitutoyo, Spain). Samples were polished using a Saphir 250 A1-ECO polisher (Neurtek, Madrid, Spain) and post cured in a JP-Selecta oven (Barcelona, Spain). Mixing was performed using a Hauschild high speed orbital mixer (Haushild, Hamm, Germany). For the ultrasonic measurements, an ultrasonics, pulser-receiver: DPR300 (JSR Ultrasonics, Pittsford, NY, USA), an oscilloscope (Tektronix, DPO5054, Tektronix, Beaverton, OR, USA), and one pair of flat water immersion wide band transducers centered at 3.5 MHz (Olympus V383-SU, 3.5 MHz, Olympus, Allentown, PA, USA) were used.

### 2.2. Experimental Methods

#### 2.2.1. Fabrication of Composite Samples

The key elements in order to establish the fabrication route for these composites are: (i) the capability to produce from moderate to highly loaded composites with a good mixture of components, complete curing of the sample, good adhesion between matrix and particles, and without internal cavities or air trapped; (ii) the compatibility of this process with the transducer manufacturing process (this means to avoid high temperatures, pressures and use of solvents that could affect the piezoelectric material and the electrical connections at the piezoelement and to ensure an interface between the piezo and the composite free of defects and with good adhesion).

Conventional approaches using vacuum degassing or ultrasonic cavitation baths are, in most cases, not efficient with very high particle loads as the viscosity of the mixture becomes very large. In this paper, we propose to fabricate the composite materials by simultaneously mixing and degassing the mixture using a high-speed orbital mixer. The mixing/degassing protocol took place during 3 mins with two stages; first stage with a velocity of 1800 rpm for 2 mins and then, a second stage where velocity was increased (10 s ramp) up to 2500 rpm for the rest of the process. This process was revealed to be fully compatible with transducer manufacturing (i.e., direct fabrication of the composite on the piezoelectric element), is efficient in achieving high particle loads without trapped air, and has good mixing, good homogeneity, and no curing problems. In addition, as the processing times are reduced (3 mins), this also allows for the use of polymers with reduced pot life that cannot be used when large degassing times (as in vacuum degassing) are required.

**Figure 2:**
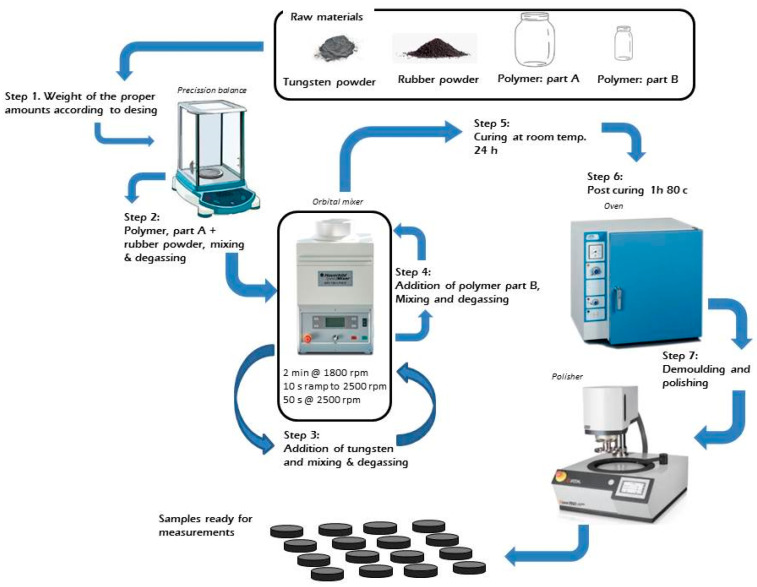
Schematic diagram of the fabrication procedure.

First, component A of the polymer is mixed with the desired particle load. The rubber is first added and mixed/degassed, then the tungsten is added and then mixed/degassed again. After this, component B of the polymer is added, and mixed and degassed. After mixing, samples were cured for 24 h at 25 °C in a cylindrical mold, then demolded and post cured for 1 h at 80 °C in a JP-Selecta oven (JPS, Barcelona, Spain). To avoid any manipulation of the sample after mixing, the cylindrical mold is the same recipient that was used for mixing. Once the samples cooled down, they were demolded and polished, using an automatic Saphir 250 A1-ECO polisher (Neurtek, Madrid, Spain), to achieve uniform, flat and parallel surfaces. Thickness variation for each sample was kept under 200 μm. Polished surfaces of the composites were observed with an optical microscope to verify the dispersion of the fillers. The fabrication route is also shown in Figure 2.

As a first and simple verification of the fabrication process, the density of the samples was measured and compared with the nominal density estimated from the amount of the different materials added to the mixer. In addition, the measured ultrasonic velocity and attenuation and comparison with theoretically predicted values are also used to detect problems like poor compatibility between filler and matrix, the presence of trapped air or lack of homogeneity. To test the homogeneity of the samples, in a few cases, thicker disks were fabricated and cut into two thinner disks that were polished and measured to determine if there is any gradient of properties. No significant differences were observed. In addition, no curing problems were observed in any of the cases presented here. All fabricated samples could be machined and polished. 

#### 2.2.2. Ultrasonic Measurements

**Figure 3:**
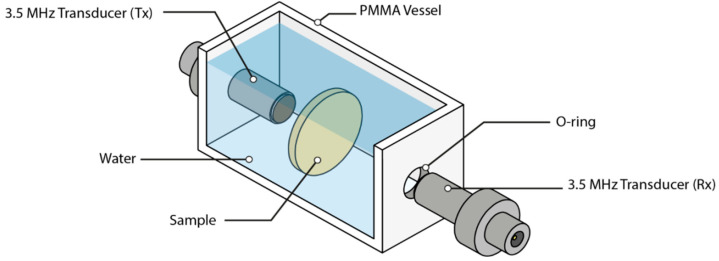
Ultrasonic water-immersion through-transmission layout.

Ultrasonic measurements were performed using a pulser-receiver (JSR Ultrasonics, DPR300, Pittsford, NY, USA), an oscilloscope (Tektronix, DPO5054, Tektronix, Beaverton, OR, USA), one pair of flat-water immersion wide band transducers centered at 3.5 MHz (Olympus V383-SU, 3.5 MHz Olympus, Allentown, PA, USA) and a custom made tank (45 × 45 × 120 mm^3^ PMMA) (see Figure 3) that easily allows for transducers and sample positioning. 

All measurements were performed in distilled and degassed water at 22 °C. Samples were first immersed in water and vacuum degassed to ensure that no air bubble is trapped on the sample surface, as this will strongly affect the measured attenuation. Then, temperature was stabilized at 22 °C and measurements performed. All measurements were performed at normal incidence. The through transmitted signal without a sample in between is used as reference or calibration. Then, the sample is put in between the transducers. Acquired signals were transferred to MATLAB where Fast Fourier Transform (FFT) was extracted to obtain the magnitude and phase spectra of the transmission coefficient. Samples were measured at several points (up to 5) to verify the homogeneity of the samples. Phase spectra allow the determination of the velocity in the sample if sample thickness and velocity in the water are known [44,45], while magnitude spectra permit the determination of attenuation and variation of the attenuation with the frequency if impedance of the water and attenuation in the water are known [45]. In addition, the variation in the attenuation coefficient with the frequency is quantified using a power law (Equation (5))

(5) $$\alpha =\alpha_{0}{\left(\frac{f}{f_{0}}\right)}^{n},$$

#### 2.2.3. Other Measurements

Weight (precision analytical laboratory balance, Nahita Blue), diameter (caliber) and thickness (micrometer, Mitutoyo) of all samples were measured and material density worked out. As the density of a composite material ($\rho_{comp}^{}$) can also be obtained from the density of its i-constituents ($\rho_{i}$) and their volumetric fraction ($\phi_{i}$) (Equation (2)), the agreement between measured and calculated density can be used to verify that the proper proportions of components were effectively added, their correct mixture during fabrication and the lack of any trapped air. In this comparison, it is considered that the main source of error is the variability of the sample thickness.

### 2.3. Theoretical Methods: Moelling Composite Properties

The purpose of the composite modelling is to make possible the prediction of the properties of the composites when the properties of the constituent materials and the volume fraction of the components are known. In this particular case, we are interested in the capability to predict the composite impedance, the ultrasonic attenuation coefficient and its variation with the frequency. So far, efforts have been focused on models that permit the calculation of the elastic moduli of two-phase composites from the elastic moduli of constituent materials and the volume fraction. With these moduli and the composite effective density, it is possible to work out the ultrasound velocity and the acoustic impedance. Reviews of the different modelling approaches can be seen in [16,22,42,46]. The simplest approach is the use of averaging models or mixture rules like the well-known Voigt and Reuss models (or series and parallel model). The main advantage is that these models are very simple and provide predictions for the entire volume fraction range. They provide an upper and lower limit for the composite properties depending on how the two phases are distributed in the space. The main drawback of these mixture rules is that when properties of the two constituent phases are very different (for example epoxy resin and tungsten), the upper and lower bounds are too separated such that they have no predictive value. The approach of Hashin and Shtrikman provides an improvement as upper and lower bounds are closer [46,47]. However, when composite components are too different, the separation between these two limits is still too big and they may have little predictive value [17,46].

According to the Hashin-Shtrikman (HS) approach [47] for a two-phase composite, the modulus of compressibility (*K_comp_*) and stiffness (*G_comp_*) of the composite are given by (Equations (6) and (7)):

(6) $$K_{comp}^{L,U}=K_{1,2}+\frac{v_{2,1}}{1/\left(K_{2,1}-K_{1,2}\right)+3v_{1,2}/\left(3K_{1,2}+4G_{1,2}\right)},$$

and

(7) $$G_{comp}^{L,U}=G_{1,2}+\frac{v_{2,1}}{1/\left(G_{2,1}-G_{1,2}\right)+6\left(K_{1,2}+2G_{1,2}\right)v_{1,.2}/\left[5G_{1,2}\left(3K_{1,2}+4G_{1,2}\right)\right]},$$

where the subscripts 1 and 2 refer to the two components and *v* is the volume fraction, *K*_2_ > *K*_1_ and *G*_2_ > *G*_1_ and superscripts *L* and *U* stand for the Upper and the Lower limits of the Hashin-Strikman model. In particular, *L* is the exact solution for the composite made of matrix of phase “one” material in which spherical inclusions of phase “two” material are distributed in a particular way. In addition, *U* is the exact solution for matrix of phase “two” material in which spherical inclusions of phase “one” material are distributed in a particular way.

The coherent potential approximation (CPA) model is based on the scattering theory. This model predicts the modulus of compressibility (*K_comp_*) and stiffness (*G_comp_*) of two phase composites, assuming that the inclusions are spherical, the wavelengths are much longer than the size of the inclusions, and multiple scattering effects are negligible (Equations (8)–(10)):

(8) $$\frac{1}{K_{comp}+\frac{4}{3}G_{comp}}=\frac{v_{1}}{K_{1}+\frac{4}{3}G_{comp}}+\frac{v_{2}}{K_{2}+\frac{4}{3}G_{comp}},$$

(9) $$\frac{1}{G_{comp}+F}=\frac{v_{1}}{G_{1}+F}+\frac{v_{2}}{G_{2}+F},$$

(10) $$F=\frac{G_{comp}}{6}\;\frac{9K_{comp}+8G_{comp}}{K_{comp}+2G_{comp}},$$

where subscripts 1 and 2 refer to the matrix and the inclusions, respectively, and *v* is the volume fraction.

In order to predict both the attenuation coefficient and the acoustic impedance of two-phase composites we make use of the correspondence principle of viscoelasticity, where complex elastic modulus (K*, G*) are used to take into account the dynamic damping in the material (Equation (11)):

(11) $$K\to K^{*},\;\;G\to \;G^{*},$$

Complex wave number for longitudinal and shear waves is defined from the angular frequency (*ω*), and the attenuation coefficient ($\alpha_{L,S}$), (Equation (12)):

(12) $$k_{L,S}^{*}=\frac{\omega }{v_{L,S}}-i\alpha_{L,S},$$

where the subscript L and S denote longitudinal and shear wave, respectively. The complex wave velocity ($v_{L,S}^{*}$) is given then by (Equation (13)):

(13) $$v_{L,S}^{*}=\frac{\omega }{k_{L,S}^{*}},$$

and complex elastic moduli ($K^{*},$ $G^{*}$) are obtained from (Equation (14))

(14) $$K^{*}+\frac{4}{3}G^{*}={\left(v_{L}^{*}\right)}^{2}\rho_{comp},\;G^{*}={\left(v_{S}^{*}\right)}^{2}\rho_{comp},$$

These complex elastic moduli of the composite components can be used with Equations (6) and (7) or (8)–(10) to calculate the complex elastic moduli of the composite and hence the attenuation coefficient. This can be repeated for several frequencies, so that variation in the attenuation coefficient with the frequency can be calculated. This method to calculate the attenuation coefficient in the composite takes into account the value of attenuation in each component and the volume fraction, but not the contribution of scattering losses. In this sense, this prediction can be expected to be more accurate in the case of epoxy resins loaded with rubber particles, as the scattering of the rubber particles in the epoxy matrix is expected to be reduced, but the increase in the attenuation due to the attenuation in the rubber is expected to be large. On the contrary, actual attenuation can be expected to be larger than the calculated one in tungsten-loaded epoxy resins as the attenuation in the tungsten is expected to be reduced but the contribution of the scattering can be expected to be significant.

In order to predict both the attenuation coefficient and the acoustic impedance of three phase composites, we propose the following approach. First, we model the composite made of epoxy resin and micronized rubber. As elastic moduli and density of these two materials are not very different, the Hashin-Strikman approach can be used to get a sensible prediction of the composite properties. In addition, we also calculate composite properties using the CPA model. Then, we consider this composite as the matrix material and we add the tungsten particles. We apply the H-S and the CPA approach to calculate the properties of this three-phase composite.

## 3. Results

The characterization of the raw materials verifies the work hypothesis (see Table 3): the rubber sample presents a very high attenuation coefficient (16.4 dB/mm at 3 MHz), compared with epoxy resin (0.7 dB/mm at 3 MHz) and polyurethane (1.3–2.7 dB/mm at 3 MHz) and also a lower variation with frequency (n = 0.79), which is good to retain high damping at low frequencies. 

In order to first test the efficiency of the fabrication technique, the expected and the actual density of the samples are always calculated as well as the deviation between them. The main source of error in the estimated density is due to thickness variability of the samples (within 200 μm). Density error due to this thickness variability is, for example, about 3% for rubber-loaded resin samples and about 6% for tungsten-loaded epoxy resin samples. Density deviations in Table 5 and Table 6 can be explained by these errors. On the contrary, some of the tungsten-loaded polyurethane samples in Table 7 (−7.5% and −11.7%), present a larger deviation that, most likely, suggests the presence of some trapped air in the composite or some mixing deficiencies. 

**Figure 4:**
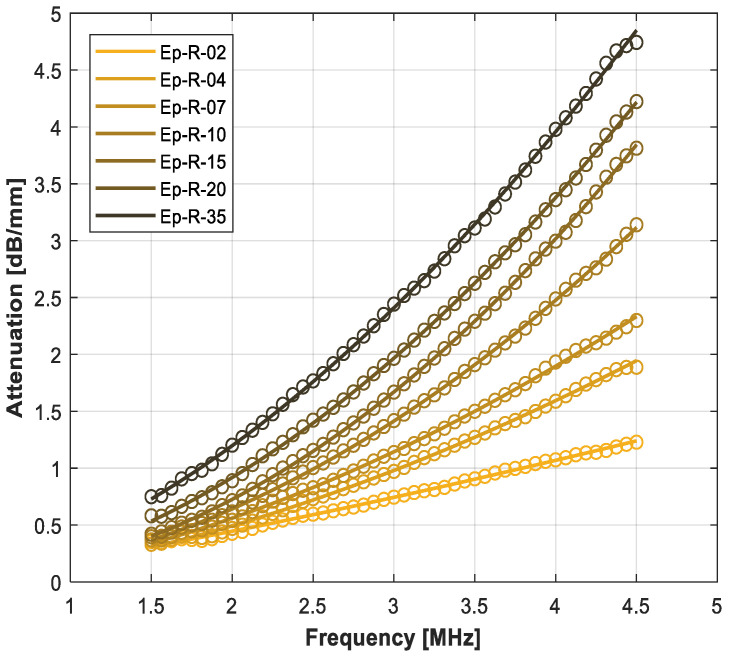
Variation in the ultrasonic attenuation coefficient with the frequency for the rubber-loaded epoxy resin composites. Open circles: measurements. Solid line: fitting using a power law (Equation (1)).

**Figure 5:**
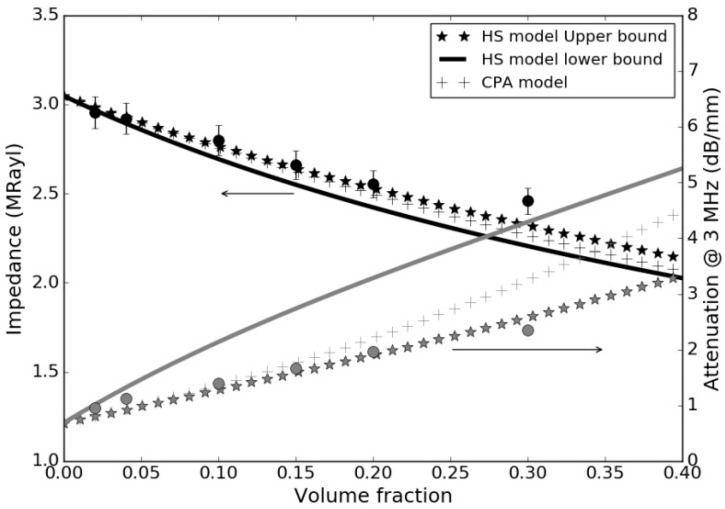
Variation in the impedance (black) and the ultrasonic attenuation coefficient at 3 MHz (gray) with the rubber volume fraction in the rubber-loaded epoxy composites. Circles: experimental measurements. Solid line: HS model, lower bound; Starts: HS, upper bound, +: CPA model.

Table 9 summarizes the measured ultrasonic properties (velocity, attenuation at 3 MHz and variation with the frequency assuming a power law) for the rubber-loaded epoxy composites. Figure 4 shows the measured variation in the attenuation coefficient with the frequency, and Figure 5 shows the comparison between the measured and the calculated acoustic impedance and attenuation coefficient at 3 MHz in the micronized-rubber-loaded epoxy composites (using the upper and lower HS bounds and CPA models).

**Figure 6:**
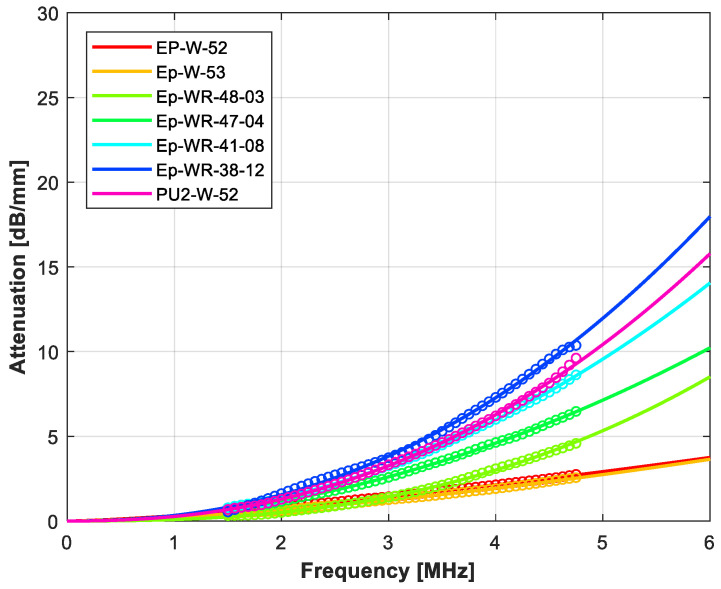
Variation in the ultrasonic attenuation coefficient with the frequency for the rubber + tungsten-loaded epoxy resin composites. High impedance series. Open circles: measurements. Solid line: fitting using a power law.

**Figure 7:**
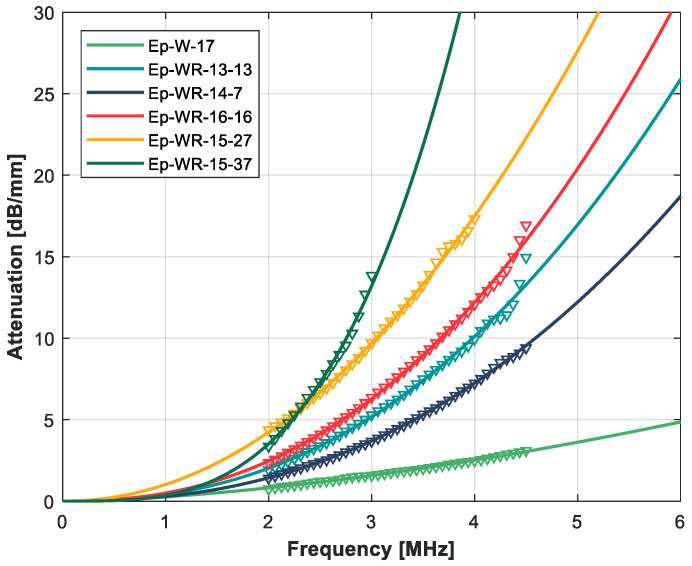
Variation in the ultrasonic attenuation coefficient with the frequency for the rubber + tungsten-loaded epoxy resin composites. Low impedance series. Open circles: measurements. Solid line: fitting using a power law.

**Figure 8:**
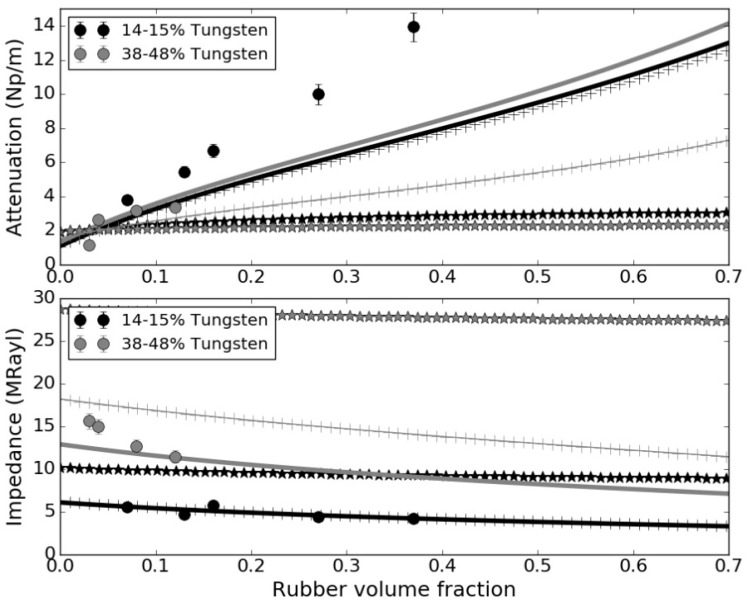
Attenuation (**up**) and Impedance (**bottom**) vs. rubber volume fraction. Gray: tungsten volume fraction: 38–48%. Black: tungsten volume fraction 14–15%. Circles: Experimental measurements; Solid line: HS model, lower bound; Starts: HS model, upper bound; +: CPA model.

Table 10 presents the measured impedance, and ultrasound velocity and attenuation for the rubber + tungsten-loaded epoxy composites, Figure 6 and Figure 7 show the variation in the attenuation coefficient with the frequency, and Figure 8 presents measured and calculated (upper and lower HS bounds and CPA models) attenuation and impedance vs. rubber volume fraction. For theoretical calculations, it is assumed that tungsten volume fraction is either 45% or 14%.

**Figure 9:**
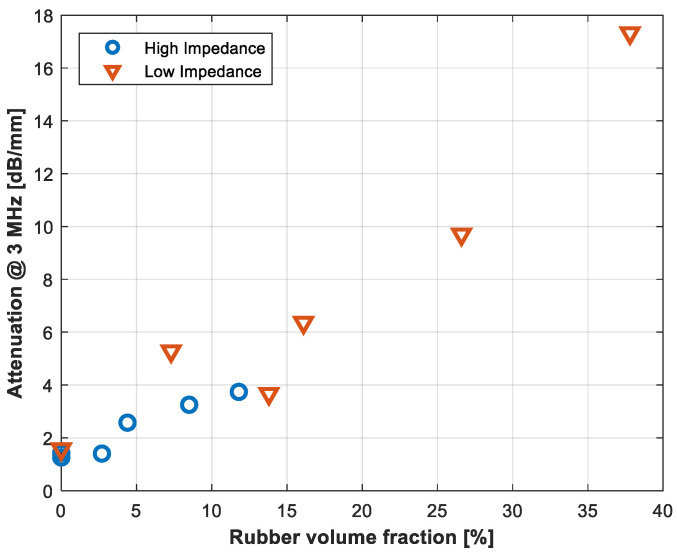
Ultrasonic attenuation coefficient at 3 MHz vs. rubber volume fraction. Results for both the high and the low impedance series.

Finally, Figure 9 shows the variation in the ultrasonic attenuation at 3 MHz with rubber volume fraction for tungsten + rubber + epoxy composite (for both the low and the high impedance series).

For completeness, Table 11 and Table 12 show the measured properties of the tungsten-loaded epoxy and polyurethane, respectively.

## 4. Discussion

As a first test of the capability of the rubber powder to increase the ultrasonic attenuation coefficient, samples with rubber volume fraction from 2 to 35% were fabricated and measured. As expected, the rubber load produces a moderate decrease of the acoustic impedance (up to 22%), and a remarkable increase in the attenuation coefficient (up to 236%). Compared with the unloaded epoxy, the variation with frequency changes notably (from n = 0.9 to n~1.8), probably due to the contribution of either the scattering produced by the rubber particles or their viscoelastic response. Compared with attenuation in polyurethane (Table 3), this approach permits the achievement of similar attenuation values but a wider range of variation and allows for a precise tailoring of the attenuation in the composite. As expected, the difference in the predictions made with HS upper and lower bounds are reduced (see Figure 5). The HS-UB model provides the closest predictions to the experimental data for both impedance and attenuation.

As it is already well known, loading the polymer with tungsten powder permits the increase of the acoustic impedance of the composite. This result is also reproduced here, see data in Table 11 and Table 12. Compared with epoxy resin, the use of polyurethanes provides composites with relatively lower acoustic impedance, mainly due to the lower acoustic impedance of the polyurethanes, which is a negative feature for backing blocks. In addition, in some cases, the larger viscosity or shorter pot life time of the polyurethane complicate the composite mixing, and this limits the maximum possible load of particles, so this also contributes to further reduce the impedance of these composites. For tungsten-loaded epoxy and polyurethane composites (Table 11 and Table 12), the measured impedance is close to the impedance predicted by the HS-LB model, with the exception of PU1-W-45 that presents a much smaller value, that is outside the HS bounds, so these suggest the presence of some air trapped.

Measurements also confirm that by loading the polymer with tungsten particles, the attenuation increases. The HS-LB model also provides good predictions for the attenuation in the composites with the exception of PU1 composites where measured attenuation is much larger than expected. This can be due to the presence of small air bubbles.

For the proposed solution for an intermediate impedance backing block with enhanced attenuation (epoxy + rubber + tungsten, impedance 5–7 MRayl), the measurements confirm the possibility to significantly increase the attenuation in the composite material by adding a third phase of rubber powder. As the required load of tungsten is reduced (about 13–15%), a higher load of rubber powder is possible (up to 37%). Measurements reveal that the attenuation coefficient can be increased (from 1.44 dB/mm, up to 13.95 dB/mm) with a moderate impedance reduction (from 6.7 to 4.2 MRayl). Comparison with calculated values (Figure 8) reveals that measured attenuation is larger than expected while the impedance is very close to the HS-LB approach. This behavior can be the result of the contribution of the scattering of the particles or can be due to a poor bounding between the particles and the matrix. On the other hand, for the proposed solution for a high impedance backing with enhanced attenuation (epoxy + rubber + tungsten, impedance 10-15 MRayl), the measurements confirm the possibility to increase, in a moderate way, the attenuation in the composite material by adding a third phase of rubber powder. As the required load of tungsten is larger (about 30–50%), only a smaller load of rubber powder is possible in this case (up to 12%). Measurements reveal that the attenuation coefficient can be increased (from 1.4 dB/mm up to 3.4 dB/mm) while the impedance decreases (from 18 to 12 MRayl). In this case, the predicted attenuation using the HS-LB provides a good matching into the measured values, while the measured impedance values are within the CPA and the HS-LB model predictions. 

Table 13 shows a comparison of attenuation and impedance values obtained for rubber + tungsten-loaded epoxy and main reference values obtained from previous published works. The largest attenuation value is obtained for polyurethane composites (13–15 dB/mm) [37], though this approach is only valid for very low impedance materials (<3.5 MRayl). Very similar values are obtained in this work (13.95 dB/mm) but in this case with a larger impedance (4.17 MRayl), which make this approach more interesting for backing blocks. For high impedance materials (>10 MRayl), the best solution is the one provided in this work while for intermediate impedance materials (5–10 MRayl), the solution of this paper provides similar results to that in [44].

**Table 13:** Comparative of attenuation coefficient and acoustic impedance values in particle-loaded polymer composites.

Reference	Attenuation Coeff @ 3 MHz (dB/mm)	Acoustic Impedance (MRayl)
Lutsch [27]	2.4	~8
Tiefensee et al. [36]	5	4-7
State et al. [37]	13–15 7.1	2.6–3.3
Cho et al. [40]	3–5	4–5
Nguyen et al. [42]	10	8.7
	1.8	11.7
Abas et al. [32]	1	7
Nguyen et al. [46]	9	8.7
El-Tantawy &. Sung [41]	2.2–3.9	2–7.8
Wang et al. [48]	~5.0	3–5.5
This work		
Rubber + epoxy	0.96–2.35	2.3–2.9
Tungsten + epoxy	1.4–1.5	6.8–18.5
Tungsten + polyurethane	4–27	6.9–10.5
Rubber + Tungsten (low) + epoxy	4–14	4–5
Rubber + Tungsten (high) + epoxy	1.2–3.4	12–16

## 5. Conclusions

This work presented a study of the possibilities to control the damping in tungsten-loaded polymers for backing blocks in ultrasonic transducers by adding micronized rubber powder obtained from end-of-life tires (ELT). A fabrication technique based on a high-speed orbital mixer is proposed as it is compatible with transducers’ fabrication restrictions and allows in situ fabrication and curing of the backing block directly on the piezoelectric element, so that interface problems can be minimized. It can be concluded that is it possible to fabricate composites in the impedance range of 4–5 MRayl with attenuation coefficient in the range 4–14 dB/mm (at 3 MHz), and higher impedance composites (12–16 MRayl), with attenuation in the range 1.2–3.4 dB/mm. In fact, it has been shown that the attenuation coefficient in the composite increases almost linearly with the load of rubber regardless of the load of tungsten (Figure 9).

The inclusion of rubber powder in tungsten-loaded polymers permits the tailoring of several functionalities of the composite. In the case of materials for ultrasonic transducers these functionalities comprise the capability to simultaneously and independently control the impedance, ultrasound velocity, ultrasound attenuation, variation of ultrasound attenuation with the frequency, the scattering strength and even the EMI shielding. In particular, it is possible to obtain materials with acoustic impedance in the range 2.35–15.6 MRayl, ultrasound velocity in the range 790–2570 m/s, attenuation at 3 MHz, from 0.96 up to 27 dB/mm with a variation of the attenuation with the frequency following a power law with exponent in the range 1.2–3.2. In addition, the inclusion of these materials in piezoelectric transducers, as backing blocks or matching layers, permits the further control of different properties and functions of the transducer, such as the bandwidth, the ringing, the amplitude and time for the appearance of the backing block back echo and the sensitivity.

For the fabrication of backing materials for ultrasonic piezoelectric transducers based on ceramics and single crystals, the best results correspond to Ep-WR-48-03, Ep-WR-47-04, Ep-WR-41-08 and Ep-WR-38-12 that combine large impedance (good damping that contributes to enlarge the bandwidth, reducing the ringing and improving axial resolution) and high attenuation and low velocity (that contribute to eliminate the backing block back echo). For the fabrication of backing materials for ultrasonic piezoelectric transducers based on 1-3 composites (ceramic volume fraction between 30 and 70%), the best option can be Ep-WR-15-27 and Ep-WR-15-37 that combine moderate impedance, large attenuation and low ultrasonic velocity.

Therefore, the materials and the manufacturing route proposed provide an interesting alternative to tailor the properties of ultrasonic transducers’ backing blocks. In addition, this solution also offers an application for the use of micronized rubber recycled from ELT. 

## 6. Patents

Title: “A manufacturing method of a passive composite material for an ultrasonic transducer”. Number: P202230611. Date: 5 July 2022.

Title: “Use of composite material as an artificial tissue or organ for testing the performance of an ultrasound diagnosis apparatus”. Number: P202230610. Date: 5 July 2022.

## Figures and Tables

**Table 1:** Raw materials.

Material Description	Commercial Name	Supplier	Other Info
Epoxy resin	EpoxAmite 101	SmoothOn (Macungie, PA, USA)	Viscosity: 1000 cps Pot life: 11 min
Polyurethane resin	Urebond	SmoothOn (PA, USA)	Viscosity: 5400 cps Pot life: 5 min
Polyurethane resin	Liquid Plastic	SmoothOn (PA USA)	Viscosity: 80 cps Pot life: 3 min
Tungsten powder	--	Alfa Aesar (Haverhill, MA, USA)	Tungsten 99.9% Particle size 12 μm
Cryogenic rubber powder	MicroDyne 75-TR	Lehigh Technologies (Atlanta, GA, USA)	Particle size < 70 μm (90% wt has a smaller size than 70 μm

**Table 2:** Disk samples (30 mm diameter) of raw materials for ultrasonic characterization.

Sample Denomination	Material	Thickness (mm)
Ep-00	EpoxAmite	4.25 ± 0.13
PU1-00	Urebond	4.82 ± 0.15
PU2-00	Liquid Plastic	4.45 ± 0.13
Rb-00	Rubber	2.73 ± 0.14

**Table 3:** Ultrasonic properties of the raw materials.

Sample	Density (kg/m^3^)	Ultrasound Velocity (m/s)	α @ 3 MHz (dB/mm)	n
Ep-00	1150 ± 33	2620 ± 75	0.69 ± 0.02	0.90
PU1-00	1125 ± 32	1700 ± 50	1.34 ± 0.04	1.13
PU2-00	1130 ± 30	2195 ± 66	2.66 ± 0.08	1.11
Rb-00	1100 ± 50	1200 ± 60	16.43 ± 0.80	0.79

**Table 4:** Properties of the Tungsten powder.

Property	Value
Longitudinal velocity (m/s)	5200
Bulk modulus (GPa)	305
E (GPa)	414
Longitudinal attenuation @ 5 MHz (dB/m)	66.5
“*n*”	1.0
Shear Modulus (GPa)	162.3
Density (kg/m^3^)	19,300

**Table 5:** Rubber-powder-loaded epoxy resin composites.

Sample Denomination	Rubber Volume Fraction (%)	Sample Thickness (mm)	Density Deviation (%)
Ep-R-02	2	4.5 ± 0.13	−0.32
Ep-R-04	4	4.1 ± 0.12	−0.39
Ep-R-10	10	3.9 ± 0.12	−2.32
Ep-R-15	15	4.7 ± 0.14	−2.49
Ep-R-20	20	4.2 ± 0.13	−4.39
Ep-R-30	30	3.9 ± 0.12	−1.92
Ep-R-35	35	4.1 ± 0.12	−0.32

**Table 6:** Tungsten powder + rubber-powder-loaded epoxy resin composites.

	Sample Denomination	Tungsten/Rubber Volume Fraction (%)	Sample Thickness (mm)	Density Deviation (%)
Low W load	Ep-WR-14-7	14.1/7.3	2.96 ± 0.14	−2.44
	Ep-WR-13-13	13.9/13.8	2.82 ± 0.16	−3.84
	Ep-WR-16-16	15.9/16.1	2.67 ± 0.15	−1.73
	Ep-WR-15-27	15.2/26.6	3.27 ± 0.18	−2.93
	Ep-WR-15-37	15.2/37.8	4.35 ± 0.27	−4.35
High W load	Ep-WR-48-03	48/3.2	3.8 ± 0.24	−1.54
	Ep-WR-47-04	47.1/4.4	3.2 ± 0.21	−2.38
	Ep-WR-41-08	41.1/8.3	1.8 ± 0.15	−1.62
	Ep-WR-38-12	38.1/12.5	4.0 ± 0.26	−1.97

**Table 7:** Samples of tungsten-loaded epoxy resin composites.

Sample Denomination	Tungsten Volume Fraction (%)	Sample Thickness (mm)	Density Deviation (%)
Ep-W-16	16.0	2.4 ± 0.12	−5.32
Ep-W-52	52.2	2.6 ± 0.13	2.81
Ep-W-53	53.2	2.7 ± 0.13	−3.55

**Table 8:** Samples of tungsten-powder-loaded polyurethane composites.

Sample Denomination	Tungsten Volume Fraction (%)	Sample Thickness (mm)	Density Deviation (%)
PU1-W-45	45.9	1.88 ± 0.11	−7.5
PU1-W-37	37.0	3.9 ± 0.23	1.7
PU2-W-52	51.7	2.63 ± 0.16	−11.7
PU2-W-37	37.0	4.8 ± 0.29	−3.0

**Table 9:** Measured ultrasonic properties of the rubber-loaded epoxy composites.

Sample	Longitudinal Ultrasound Velocity (m/s)	Impedance (MRayl)	α @ 3 MHz (dB/mm)	n
Ep-R-02	2567 ± 77	2.94 ± 0.09	0.96 ± 0.03	1.7
Ep-R-04	2536 ± 75	2.90 ± 0.09	1.12 ± 0.03	1.8
Ep-R-10	2423 ± 70	2.71 ± 0.08	1.39 ± 0.04	1.9
Ep-R-15	2298 ± 67	2.56 ± 0.08	1.66 ± 0.05	2.0
Ep-R-20	2202 ± 66	2.40 ± 0.07	1.97 ± 0.06	1.9
Ep-R-35	2111 ± 62	2.35 ± 0.07	2.35 ± 0.07	1.7

**Table 10:** Measured ultrasonic properties of the tungsten + rubber-loaded epoxy composites.

	Sample	Longitudinal Ultrasound Velocity (m/s)	Impedance (MRayl)	α @ 3 MHz (dB/mm)	n
Low W-load	Ep-WR-14-7	1562 ± 94	5.57 ± 0.3	3.80 ± 0.2	2.3
	Ep-WR-13-13	1311 ± 81	4.66 ± 0.3	5.46 ± 0.3	2.3
	Ep-WR-16-16	1463 ± 87	5.77 ± 0.3	6.69 ± 0.4	2.3
	Ep-WR-15-27	1166 ± 69	4.40 ± 0.3	10.01 ± 0.6	2.0
	Ep-WR-15-37	1127 ± 69	4.17 ± 0.3	13.95 ± 0.8	3.2
High W-load	Ep-WR-48-03	1618 ± 95	15.64 ± 0.9	1.18 ± 0.07	2.5
	Ep-WR-47-04	1594 ± 94	15.01 ± 0.9	2.66 ± 0.1	2.0
	Ep-WR-41-08	1507 ± 90	12.70 ± 0.9	3.19 ± 0.2	2.1
	Ep-WR-38-12	1459 ± 89	11.48 ± 0.9	3.36 ± 0.2	2.2

**Table 11:** Measured ultrasonic properties of the tungsten-loaded epoxy composites.

Sample	Longitudinal Ultrasound Velocity (m/s)	Impedance (MRayl)	α @ 3 MHz (dB/mm)	n
Ep-W-17	1695	6.77	1.44	1.7
Ep-W-52	1706	18.48	1.53	1.4
Ep-W-53	1666	17.22	1.35	1.6

**Table 12:** Measured ultrasonic properties of the tungsten-loaded polyurethane composites.

Sample	Longitudinal Ultrasound Velocity (m/s)	Impedance (MRayl)	α @ 3 MHz (dB/mm)	n
PU1-W-45	788	6.90	24.44	1.2
PU1-W-37	863	6.90	27.02	1.05
PU2-W-52	1139	10.58	3.97	2.3
PU2-W-37	1259	9.59	6.69	1.05
PU1-W-45	788	6.90	24.44	1.2

## Data Availability

Data are available upon request to the corresponding author.

## References

[1] Rothon B. (2017). Fillers for Polymer Applications.

[2] Bagheri R., Marouf B.T., Pearson R.A. (2009). Rubber-toughened epoxies: A critical review. Polym. Rev..

[3] Kargarzadeh H., Ahmad I., Abdullah I. (2015). Chapter 10: Mechanical Properties of Epoxy-Rubber Blends. Handbook of Epoxy Blends.

[4] Ismail H., Omar N.F., Othman N. (2011). Effect of carbon black loading on curing characteristics and mechanical properties of waste tyre dust/carbon black hybrid filler filled natural rubber compounds. J. Appl. Polym. Sci..

[5] Colom X., Carrillo F., Cañavate J. (2007). Composites reinforced with reused tyres: Surface oxidant treatment to improve the interfacial compatibility. Compos. Part A Appl. Sci. Manufac..

[6] Valášek P. (2014). Mechanical Properties of Epoxy Resins Filled with Waste Rubber. Manuf. Technol..

[7] Ramarad S., Khalid M., Ratnam C.T., Chua A.I., Rashmi W. (2015). Waste tire rubber in polymer blends: A review on the evolution, properties and future. Prog. Mater. Sci..

[8] Woldemariam M.H., Belingardi G., Koricho E.G., Reda D.T. (2019). Effects of nanomaterials and particles on mechanical properties and fracture toughness of composite materials: A short review. AIMS Mater. Sci..

[9] Móczó J., Pukánszky B., Palsule S. (2015). Particulate Fillers in Thermoplastics. Encyclopedia of Polymers and Composites.

[10] Lakes R.S. (2002). High damping composite materials: Effect of structural hierarchy. J. Compos. Mater..

[11] Sankaran S., Deshmukh K., Ahamed M.B., Khadheer Pasha S.K. (2018). Recent advances in electromagnetic interference shielding properties of metal and carbon filler reinforced flexible polymer composites: A review. Compos. Part A Appl. Sci. Manufact..

[12] Quesenberry M.J., Madison P.H., Jensen R.E. Characterization of Low Density Glass Filled Epoxies, Army Research Laboratory, March 2003, ATRL-TR-2938. https://apps.dtic.mil/sti/pdfs/ADA412137.pdf.

[13] Valentín J.L., Pérez-Aparicio R., Fernandez-Torres A., Posadas P., Herrero R., Salamanca F.M., Navarro R., Saiz-Rodríguez L. (2020). Advanced Characterization of Recycled Rubber from End-of-life Tires. Rubber Chem. Technol..

[14] Valentín J.L., Saiz-Rodríguez L., Pérez-Aparicio R. (2021). Guía para el empleo de caucho reciclado procedente del neumático en la industria del caucho. Rev. De Plaásticos Mod..

[15] Newnham R.E., Skinner D.P., Cross L.E. (1978). Connectivity and piezoelectric-pyroelectric composites. Mat. Res. Bull..

[16] Levassort F., Lethiecq M., Desmare R. (1999). Effective electroelastic moduli of 3-3(0-3) piezocomposites. IEEE Trans. Ultrason. Ferroelectr. Freq. Control.

[17] Gómez Alvarez-Arenas T.E., Mulholland A.J., Hayward G., Gomatam J. (2000). Wave propagation in 0-3/3-3 connectivity composites with complex microstructure. Ultrasonics.

[18] Newnham R.E., Bowen L.J., Klicker K.A., Cross L.E. (1980). Composite piezoelectric transducers. Mater. Des..

[19] Haifeng Wang K.S., Ritter T., Cao W. (2001). High frequency properties of passive materials for ultrasonic transducers. IEEE Trans. Ultrason. Freq. Control.

[20] Banno H., Saito S. (1983). Piezoelectric and dielectric properties of composites of synthetic rubber and PbTiO or PZT. Jpn. J. Appl. Phys..

[21] Gururaja T.R., Xu Q.C., Ramachandran A.R., Halliyal A., Newnham R.E. Preparation and piezoelectric properties of fired 0–3 composites. Proceedings of the IEEE 1986 Ultrasonics Symposium.

[22] Levassort F., Lethiecq M., Certon D., Patat F. (1997). A matrix method for modelling electroelastic moduli of 0–3 piezo-composites. IEEE Trans. Ultrason. Ferroelect. Freq. Control.

[23] Gómez Álvarez-Arenas T.E., Montero F., Levassort F., Lethieq M., James A., Ringgard E., Millar C.E., Hawkins P. (1998). Ceramic powder–polymer piezocomposites for electroacoustic transduction: Modeling and design. Ultrasonics.

[24] Lau S., Li X., Zhang X., Zhou Q., Shung K.K., Ji H., Ren W. High frequency ultrasonic transducer with KNN/BNT 0–3 composite active element. Proceedings of the 2010 IEEE International Ultrasonics Symposium.

[25] Hanner K.A., Safari A., Newnham R.E., Runt J. (1989). Thin-film 0–3 polymer piezo- electric ceramic composites-piezoelectric paints. Ferroelectrics.

[26] Wang F., Wang H., Song Y., Sun H. (2012). High piezoelectricity 0–3 cement-based piezoelectric composites. Mater. Lett..

[27] Lutsch A. (1962). Solid Mixtures with Specified Impedances and High Attenuation for Ultrasonic Waves. J. Acoust. Soc. Am..

[28] Lees S., Gilmore R.S., Kranz P.R. (1973). Acoustic Properties of Tungsten-Vinyl Composites. IEEE Trans. Sonics Ultrason..

[29] Ju-Zhen W. (1989). Backing Material for the Ultrasonic Transducer. USA Patent.

[30] Sayers C.M., Tait C.E. (1984). Ultrasonic properties of transducer backings. Ultrasonics.

[31] Grewe M.G., Gururaja T.R., Shrout T.R., Newnham R.E. (1990). Acoustic properties of particle/polymer composites for ultrasonic transducer backing applications. IEEE Trans. Ultrason. Ferroelect. Freq. Control.

[32] Abas A.A., Ismail D.M.P., Sani S., Noorul M., Ahmed I. Effect of Backing layer Composition on Ultrasonic Probe Bandwith. Proceedings of the RnD Seminar 2010: Research and Development Seminar.

[33] Hidayat D., Syafei N.S., Wibawa B.M., Taufik M., Bahtiar A., Risdiana R. (2020). Metal-Polymer Composite as an Acoustic Attenuating Material for Ultrasonic Transducers. KEM.

[34] Zhang W., Jia H., Gao G., Cheng X., Du P., Xu D. (2019). Backing layers on electroacoustic properties of the acoustic emission sensors. Appl. Acoust..

[35] Zhou Q., Cha J.H., Huang Y., Zhang R., Cao W., Shung K.K. (2009). Alumina/epoxy nanocomposite matching layers for high-frequency ultrasound transducer application. IEEE Trans. Ultrason. Ferroelect. Freq. Control.

[36] Tiefensee F., Becker-Willinger C., Heppe G., Herbeck-Engel P., Jakob A. (2010). Nanocomposite cerium oxide polymer matching layers with adjustable acoustic impedance between 4 MRayl and 7 MRayl. Ultrasonics.

[37] State M., Brands P.J., van de Vosse F.N. (2010). Improving the thermal dimensional stability of flexible polymer composite backing materials for ultrasound transducers. Ultrasonics.

[38] Cafarelli A., Verbeni A., Poliziani A., Dario P., Menciassi A., Ricotti L. (2017). Tuning acoustic and mechanical properties of materials for ultrasound phantoms and smart substrates for cell cultures. Acta Biomater..

[39] Culjat M.O., Goldenberg D., Tewari P., Singh R.S. (2010). A review of tissue substitutes for ultrasound imaging. Ultrasound Med. Biol..

[40] Cho E., Park G., Lee J.-W., Cho S.-M., Kim T., Kim J., Choi W., Ohm W.-S., Kang S. (2016). Effect of alumina composition and surface integrity in alumina/epoxy composites on the ultrasonic attenuation properties. Ultrasonics.

[41] El-Tantawy F.M., Sung Y.K. (2004). A novel ultrasonic transducer backing from porous epoxy resin-titanium-silane coupling agent and plasticizer composites. Mater. Lett..

[42] Nguyen N.T., Lethiecq M., Karlsson B., Patat F. (1996). Highly attenuative rubber modified epoxy for ultrasonic transducer backing applications. Ultrasonics.

[43] Thomas R., Ahmad I., Ahmad S., Koshy S., Thomas S., Chan C.H., Pothan L., Rajisha K.R., Maria Hanna J. (2014). Blends and IPNs of Natural Rubber with Thermo-Setting Polymers. Natural Rubber Materials.

[44] Sachse W., Pao Y.H. (1978). On the determination of phase and group velocities of dispersive waves in solids. J. Appl. Phys..

[45] Kline R.A. (1984). Measurement of attenuation and dispersion using an ultrasonic spectroscopy technique. J. Acoust. Soc. Am..

[46] Nguyen T.N., Lethiecq M., Levassort F., Pourcelot L. (1996). Experimental verification of the theory of elastic properties using scattering approximations in (0-3) connectivity composite materials. IEEE Trans. Ultrason. Ferroelectr. Freq. Control.

[47] Hashin Z., Shtrikman S. (1963). A variational approach to the theory of the elastic behavior of multiphase materials. J. Mech. Phys. Solids.

[48] Wang H., Ritter T.A., Cao W., Shung K.K. Passive materials for high frequency ultrasound transducers. Proceedings of the SPIE Conference on Ultrasonic Transducer Engineering.

